# Impedimetric Detection Based on Label-Free Immunoassay Developed for Targeting Spike S1 Protein of SARS-CoV-2

**DOI:** 10.3390/diagnostics12081992

**Published:** 2022-08-17

**Authors:** Arzum Erdem, Huseyin Senturk, Esma Yildiz, Meltem Maral

**Affiliations:** Analytical Chemistry Department, Faculty of Pharmacy, Ege University, Bornova, Izmir 35100, Turkey

**Keywords:** SARS-CoV-2 S1 protein, electrochemical immunosensors, electrochemical impedance spectroscopy, COVID-19

## Abstract

After the COVID-19 pandemic started all over the world, great importance was placed on the development of sensitive and selective bioanalytical assays for the rapid detection of the highly pathogenic SARS-CoV-2 virus causing COVID-19 disease. In this present work, an impedimetric immunosensor was developed and applied for rapid, reliable, sensitive and selective detection of the SARS-CoV-2 S1 protein. To detect the SARS-CoV-2 virus, targeting of the spike S1 protein was achieved herein by using S1 protein-specific capture antibody (Cab-S1) immobilized screen-printed electrode (SPE) in combination with the electrochemical impedance spectroscopy (EIS) technique. With the impedimetric immunosensor, the detection limit for S1 protein in buffer medium was found to be 0.23 ng/mL (equal to 23.92 amol in 8 µL sample) in the linear concentration range of S1 protein from 0.5 to 10 ng/mL. In the artificial saliva medium, it was found to be 0.09 ng/mL (equals to 9.36 amol in 8 µL sample) in the linear concentration range of S1 protein between 0.1 and 1 ng/mL. The selectivity of the impedimetric immunosensor toward S1 protein was tested against influenza hemagglutinin antigen (HA) in the buffer medium as well as in artificial saliva.

## 1. Introduction

After the beginning of COVID-19 pandemic, there were significant efforts to develop easy-to-use, fast, inexpensive and accurate diagnostic tests that could provide an advantage against this emerging threat.

The SARS-CoV-2 spike protein has two regions: S1 and S2. As the S1 region of the spike protein interacts with the host cell receptors, it permits the SARS-CoV-2 virus to enter the cell [1,2,3]. The S2 region of the spike protein is responsible for the fusion of the virus with the cell membrane of the host cell [3].

At this time, the diagnosis of the SARS-CoV-2 virus is of key importance due to the effects of the COVID-19 pandemic on all over the world, and reports on the topic [4,5,6,7,8,9,10,11,12,13,14,15] are increasingly available in the literature while COVID-19 pandemic still continues.

Electrochemical methods provide many advantages, such as competitive sensitivity, cost-effectiveness, rapidity, easy-to-use qualities and suitability for miniaturization, while allowing the point-of-care testing of many inherited and genetic diseases in comparison to conventional methods [16,17].

Electrochemical methods have been successfully and practically applied for the development of electrochemical immunosensors based on different types of electrodes; the screen-printed electrode (SPE), the carbon paste electrode (CPE) and the glassy carbon electrode (GCE) etc. [18,19,20].

Nowadays, SPEs are more preferentially used, due to advantages such as disposability and greater suitability for implementation to portable devices, being able to provide sensitive and precise analysis using a small of sample [12,21,22].

The electrochemical impedance spectroscopy (EIS) technique is used in many fields of electrochemistry, such as electrode kinetics, double-layer studies, solid-phase electrochemistry and bioelectrochemistry. It is a beneficial method for investigating the interface properties on conductive and semiconductor surfaces [23]. The use of this highly sensitive and label-free technique in the development of biosensing strategies is increasing gradually in the examination of numerous types of biochemical interactions (e.g., antigen-antibody, DNA-DNA interactions, drug-DNA interactions, aptamer-based protein, cell, etc.) [24,25,26].

Some earlier studies have been conducted regarding the implementation of EIS technique in the detection of SARS-CoV-2 [6,7,10,27,28,29,30,31,32,33,34,35,36,37]. For instance, Rahmati et al. [6] modified the surfaces of disposable SPCEs with Cu_2_O nanocubes and immobilized Protein A onto the modified electrode surface. After immobilization of the IgG antibody onto the surface of the modified electrode, the sample containing SARS-CoV-2 spike antigen was added onto the surface of electrode, and impedimetric measurement was conducted accordingly. In the study introduced by Torres et al. [7], angiotensin-converting enzyme 2 (ACE2) solution was added onto the surface of paper-based electrodes during 1.5 h immobilization time. After the immobilization step, SARS-CoV-2 virus detection was explored by EIS technique. Streptavidin-modified boron-doped polycrystalline diamond electrodes were developed for the immobilization of biotin-labeled SARS-CoV-2 S1 antibodies and then applied for impedimetric detection of the SARS-CoV-2 S1 protein [37]. However, the procedure was similar to the most of earlier reports on impedimetric immunosensors, with the authors following a time-consuming procedure for the development of the immunosensor, while using expensive and laborious electrodes.

In the present study, a label-free immunoassay was designed and combined with the impedimetric detection of spike S1 protein, and its applicability to real samples was also demonstrated. Under this objective, S1 protein-specific capture antibody (Cab-S1) was firstly attached onto the electrode surface. After dropping the sample containing spike S1 protein onto the surface, a specific immunoreaction occurred based on the antibody-antigen interaction. The sensitive and selective detection of S1 protein was explored impedimetrically in buffer and artificial saliva medium, as well as the selectivity of the label-free impedimetric immunosensor against Hemagglutinin antigen (HA). The analytical performance of the immunosensor was also reported with regards to its limit of detection (LOD), the reproducibility and the sensitivity.

## 2. Materials and Methods

### 2.1. Chemicals and Apparatus

Detailed information about SARS-CoV-2 spike S1 protein, its specific capture antibody (Cab-S1), hemagglutinin antigen (HA) and bovine serum albumin (BSA) is given in Table S1 of Supplementary Materials. The required information about each of the buffer solutions, as well as the details on the equipment and apparatus is also given in Supplementary Materials.

### 2.2. Procedure

The procedure based on the SARS-CoV-2 S1 protein-specific impedimetric immunosensor was developed through the following steps: (i) activation of the surface of the SPE; (ii) immobilization of Cab-S1 onto the electrode surface; (iii) blocking of the electrode surface; (iv) incubation of S1 protein at the electrode surface, and (v) impedimetric measurement.

**Activation of the surface of the SPE:** Electrochemical activation of the electrode surface was performed similarly to a previous study [12]. The solution containing 5 mM EDC and 8 mM NHS was prepared in PBS (50 mM, pH 7.40), as reported in earlier literature [38,39]. Chemical activation was carried out by dropping 8 µL of EDC/NHS solution onto the electrode surface, and it was kept for 60 min. After activation of the electrode surface, the electrode was rinsed with PBS (50 mM, pH 7.40) for 10 s. All steps were performed at room temperature unless otherwise stated.

**Immobilization of Cab-S1 onto the electrode surface:** 1 µg/mL Cab-S1 was prepared in PBS (50 mM, pH 7.40). An amount of 8 µL of Cab-S1 solution was pipetted onto the electrode surface and kept for 1 h. After immobilization of Cab-S1, the electrode was rinsed with PBS (50 mM, pH 7.40) for 10 s.

**Blocking of the electrode surface**: In order to block the free sites at the electrode surface, BSA was used as a blocking agent. An amount of 0.5 µg/mL BSA solution was prepared using PBS (50 mM, pH 7.40). Then 8 µL of BSA solution was pipetted onto the electrode surface and kept for 30 min. After the blocking step, the electrode was rinsed with PBS (50 mM, pH 7.40) for 10 s.

**Incubation of S1 protein at the electrode surface:** S1 protein was prepared in its different concentrations using PBS (50 mM, pH 7.40). An amount of 8 µL of S1 protein solution was pipetted onto the electrode surface and incubated for 30 min at 37 °C in a drying oven. After the incubation step, the electrode was rinsed with PBS (50 mM, pH 7.40) for 10 s.

**Impedimetric measurement:** Electrochemical impedance spectroscopy (EIS) measurement in the frequency range of 10,000–0.05 Hz was performed in redox probe solution containing 5 mM [Fe(CN)_6_]^3−/4−^ prepared in PBS (50 mM, pH 7.40) with 0.1 M KCl. Subsequently, 40 µL of redox probe solution was pipetted onto the electrode surface, and then EIS measurement was performed under the open circuit potential (OCP) with the following conditions: DC potential 0 V vs OCP and AC potential = 10 mV_RMS_.

**Figure 1:**
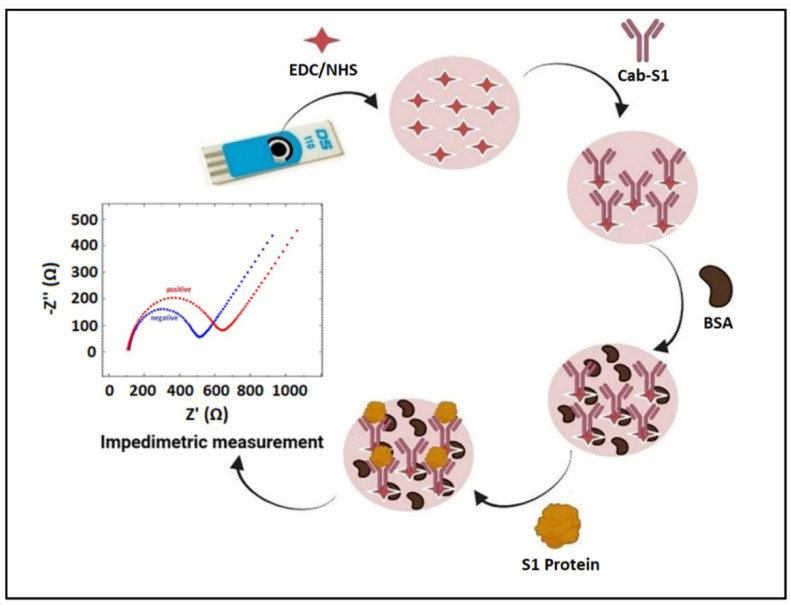
Development of label-free immunosensor with its application for impedimetric detection of spike S1 protein of SARS-CoV-2.

The schematic representation of the label-free impedimetric immunosensor specific to spike S1 protein is illustrated in Figure 1.

The selectivity of label-free immunosensor to S1 protein was analyzed against hemagglutinin antigen (HA) by following the procedure described above.

To demonstrate the applicability of the label-free impedimetric immunosensor to real samples, a batch of experiments was performed in artificial saliva medium. The samples of S1 protein in different concentrations were prepared by using diluted artificial saliva. After following each of these steps in buffer medium as established above, EIS measurements were performed.

## 3. Results & Discussion

The characterization of the construction of our immunosensor was carried out using the cyclic voltammetry (CV) technique in the redox probe solution, and accordingly, the results were given in Figure S2 and Table S2.

As shown in Figure S2, a well-defined peak was observed by unmodified screen-printed carbon electrode. A slight decrease in current was observed after chemical activation of the electrode surface with EDC/NHS. Then a decrease in current occurred after Cab-S1 immobilization onto the electrode surface. Due to the steric/conformational restriction at the electrode surface with the barrier effect of protein molecules, inhibition of electron transfer occurred, and thus a decrease in current was observed. This change in current indicates that the antibody was successfully immobilized onto the electrode surface. Then, a decrease in current with the shift in the redox peaks occurred after blocking step and then again following incubation of S1 protein on the electrode surface. An increase in ΔE_p_ was recorded from 113 mV to 132 mV after the blocking step, and similarly, an increase in ΔE_p_ (from 132 mV to 142 mV) was obtained by incubating S1 protein onto the electrode surface. Due to the insulating character of the biological components immobilized onto the electrode surface and the more steric/conformational restrictions of the proteins, the electron transfer was inhibited. Hence, a decrease in current with the shift in the redox peaks was observed, similarly to the results presented in previous studies [6,28,34,40,41].

The effects of different antibody sources upon the response was examined based on the interaction of antibodies with the spike S1 protein. The interaction of antibody-antigen was investigated using antibodies with different sources; e.g., human monoclonal antibody (mAb), rabbit mAb and rabbit polyclonal antibody (pAb), in order to perform more sensitive and specific analysis of the spike S1 protein of SARS-CoV-2. The results are shown in Table S3 of Supplementary Materials. The highest difference ratio (15.39%) was obtained in the procedure by using human mAb as Cab-S1 with the addition of spike S1 protein onto the surfaces of all electrodes. Hence, a human mAb was used as Cab-S1 for the detection of SARS-CoV-2 in our assay, similarly to earlier studies presented by Lien et al. [42] and Nessark et al. [43].

The experimental parameters, including the concentration of Cab-S1 (Table S4) and BSA (Table S5); BSA blocking time (Table S6); antigen incubation time (Table S7) and antigen incubation temperature (Table S8) were examined. The optimum experimental parameters are summarized in Table 1 with the results presented in Figure S3 of Supplementary Materials.

Next, the analytical performance of the label-free impedimetric immunosensor was explored by following the optimum experimental conditions. The diluted solutions of spike S1 protein were prepared in PBS, and EIS measurements were carried out accordingly (shown in Figure S4).

**Figure 2:**
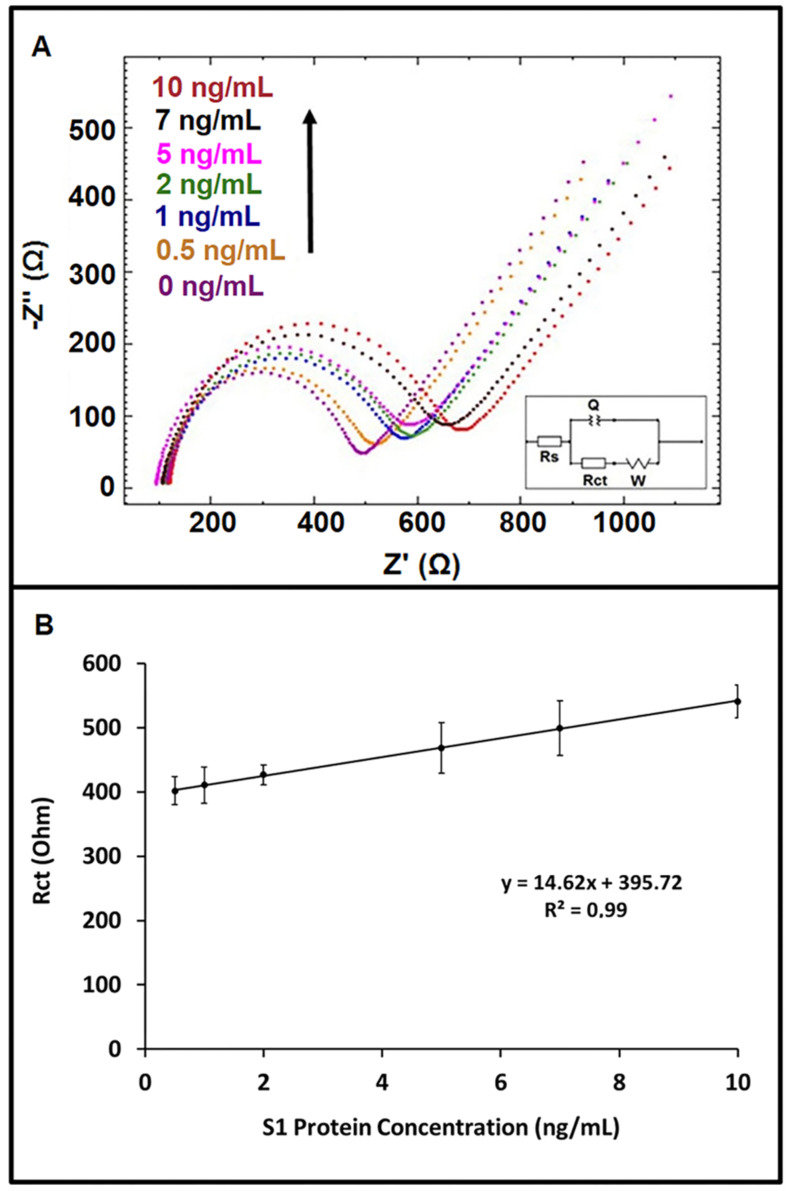
(**A**) Nyquist diagrams showing the data obtained by impedimetric immunosensor for the detection of S1 protein in its concentrations varying from 0.5 to 10 ng/mL prepared in buffer medium. (**B**) Calibration curve obtained by impedimetric detection of spike S1 protein of SARS-CoV-2 (*n* = 6).

An increase in average Rct value was recorded in the concentration range of S1 protein varying from 0.5 ng/mL to 10 ng/mL (Figure 2A). The limit of detection (LOD) is estimated by using the method of Miller and Miller [44] with a regression equation and the definition as “y = yB + 3SB” (yB is the signal of blank solution and SB is the standard deviation of the blank solution).

Based on the calibration graph with a regression equation: y=14.623x+395.72 $\left(R^{2}=0.99\right)$ (shown in Figure 2B), the LOD was estimated as 0.23 ng/mL. The sensitivity of the immunosensor was calculated by the ratio of the slope of the calibration plot (14.623) to the active surface area (0.42 cm^2^) of the working electrode, and it was found to be 34.82 Ohm mL ng^−1^ cm^−2^.

The repeatability of the impedimetric immunosensor was examined, and the results are given in Table S9. Inter-day relative standard deviation (RSD %) value was calculated by six repetitive measurements at six different concentrations of spike S1 protein of SARS-CoV-2. Accordingly, the RSD value was found to be below 10% in each concentration of S1 protein. It can be concluded that the impedimetric immunosensor specific to spike S1 protein provides precise and sensitive analysis for the detection of SARS-CoV-2.

Since the diseases COVID-19 and influenza show similar symptoms, such as cough, fever, fatigue and headache, the selectivity studies were explored herein using one of the widely tested biomarkers of influenza, HA antigen. This antigen was preferentially studied in this work, similarly to other interferents used in earlier studies developed for the detection of COVID-19; such as, influenza virus A (H1N1), 2009 influenza virus pH1N1 [4], influenza A and B antigens [6], influenza A viruses [35] (H3N2 Brisbane virus and H1N1 New Caledonia virus), influenza A virus N-protein, and influenza B virus N-protein [45], influenza B, B/Colorado [7], influenza A viruses (H1N1, H5N1, and H3N2) and influenza B [33].

**Figure 3:**
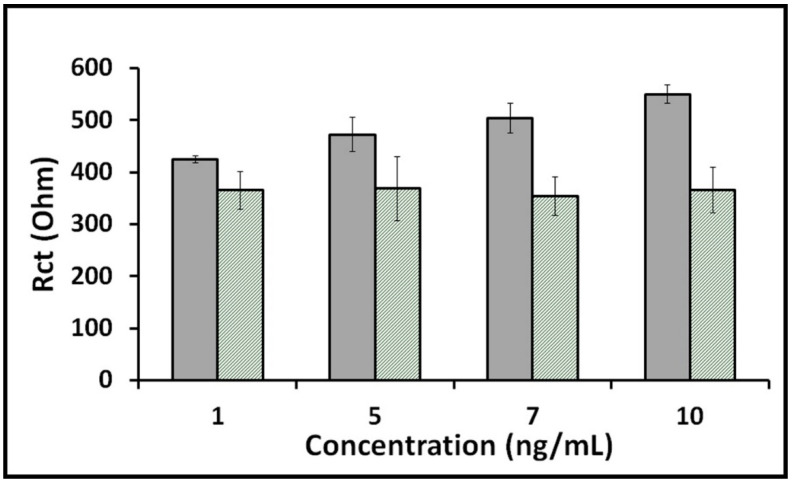
Histograms presenting the data obtained in the selectivity study performed in different concentrations of S1 protein or HA. Gray columns represent full procedure in the presence of spike S1 protein, and green-striped columns represent full procedure in the presence of HA (*n* = 3).

The selectivity studies were performed in the concentrations of 1, 5, 7 and 10 ng/mL of spike S1 protein or HA prepared in buffer medium. An increase in average Rct value was obtained while increasing the concentration of S1 protein, however, the average Rct value remained almost constant while increasing concentration of HA. (Figure 3). Even with a 10-fold increase in HA concentration (1 ng/mL to 10 ng/mL), the average Rct values remained almost constant without a significant change. However, in the presence of S1 protein, an increase in Rct value was observed proportionally to the S1 protein concentration (Figure 3). These results indicate that the impedimetric immunosensor presented a good selectivity toward its target S1 protein in contrast to HA antigen.

The implementation of the label-free impedimetric immunosensor specific to spike S1 protein of SARS-CoV-2 to real samples was investigated in the samples prepared by using artificial saliva medium, along with its application in easy and non-invasive analysis of COVID-19.

Since the artificial saliva medium is a complex matrix, it is important to minimize the effects of possible interferents available in artificial saliva medium. There are some reports in the literature [4,9,46] presenting the application of the immunosensor in undiluted artificial saliva medium as well as in diluted forms of the medium in ratios ranging from 1:5 to 1:100. Accordingly, the effect of different dilution ratio upon the immunosensor response was examined in our study.

The Rct value was measured in the presence of diluted artificial saliva medium; 1:5 and 1:20 respectively as 363.00 ± 22.63 with RSD 6.23% (*n* = 2) and 364.00 ± 5.66 with RSD 1.55% (*n* = 2). After incubation of the sample of diluted artificial saliva (1:20) onto the electrode surface, more reproducible results were obtained. The resulting Nyquist diagram is shown in Figure S5.

The difference ratio (%) was calculated according to Equation (S1) given in Supplementary materials. A higher difference ratio (30.28% increase) was found in the presence of S1 protein prepared in diluted artificial saliva in the ratio of 1:20, in contrast to the one (13.72% increase) prepared in diluted artificial saliva in the ratio of 1:5 (Figure S5).

The solutions of spike S1 protein were then prepared by using an artificial saliva dilution of 1:20, and accordingly, EIS measurements were performed after following the experimental procedure above. The data are shown in Figure S6.

**Figure 4:**
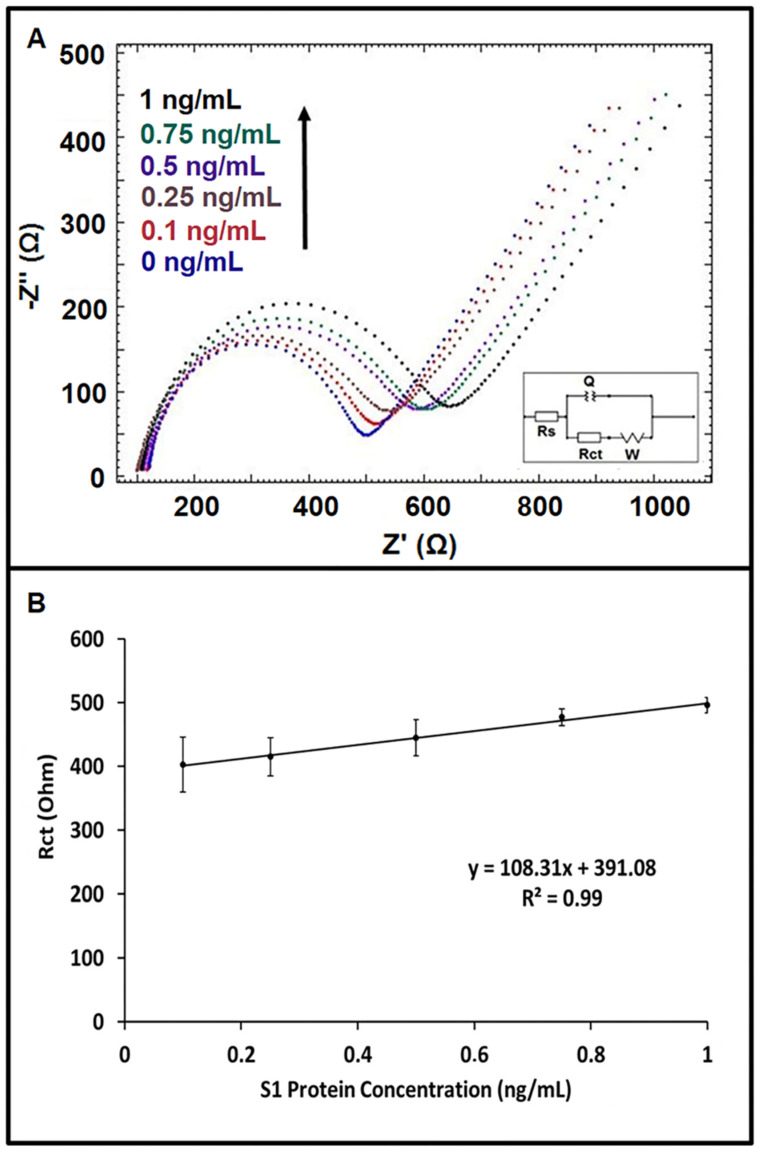
(**A**) Nyquist diagrams showing the response of the label-free impedimetric immunosensor at increasing concentrations of S1 protein in the range of 0–1 ng/mL prepared in diluted artificial saliva (1:20). (**B**) Calibration curve presenting the data for the impedimetric determination of spike S1 protein of SARS-CoV-2 in diluted artificial saliva (1:20) (*n* = 3).

An increase in average Rct value was recorded in the concentration range of 0.1 ng/mL–1 ng/mL S1 protein prepared in artificial saliva medium (Figure 4A). Based on the calibration graph with a regression equation: y=108.31x+391.08 $\left(R^{2}=0.99\right)$ (shown in Figure 4B), the LOD was found to be 0.09 ng/mL, according to the Miller and Miller method [44]. The sensitivity of the immunosensor was also calculated by the ratio of the slope of the calibration plot (108.31) to the active surface area (0.42 cm^2^) of the working electrode, and it was found to be 257.88 Ohm mL ng^−1^ cm^−2^ in artificial saliva medium.

In our study, the concentration range for S1 protein was found to be similar to the concentration range of the antigen obtained in the samples of COVID-19 patients, as investigated in saliva samples by the rRT-PCR technique [11].

**Figure 5:**
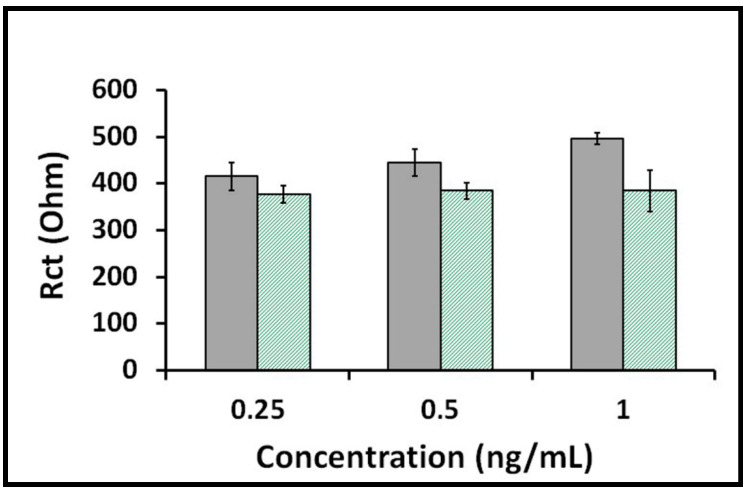
Histograms presenting the data obtained in the selectivity study performed on different concentrations of S1 protein or HA prepared in diluted artificial saliva (1:20). Gray columns represent full procedure in the presence of spike S1 protein, and green-striped columns represent full procedure in the presence of HA (*n* = 3).

Next, the selectivity of the label-free impedimetric immunosensor was explored in the medium of diluted artificial saliva (1:20). S1 protein or HA was prepared in the concentrations of 0.25, 0.5 and 1 ng/mL in diluted artificial saliva medium (1:20). A gradual increase in the average Rct was observed with increasing concentrations of spike S1 protein, whereas the average Rct remained relatively steady at increasing concentrations of HA (Figure 5). Even in a complex environment, such as artificial saliva, the immunosensor selectively detected the S1 protein prepared in three different concentrations. In addition, more repeatable results were obtained in the presence of S1 protein compared with the results observed with HA. These results indicate that the impedimetric immunosensor presented a good selectivity toward its target S1 protein over HA antigen.

A further experiment on the selectivity of the immunosensor was performed on the mixture sample of spike S1 protein and HA (1:1) prepared in diluted artificial saliva (1:20). The resulting Nyquist diagram is shown in Figure S7.

**Figure 6:**
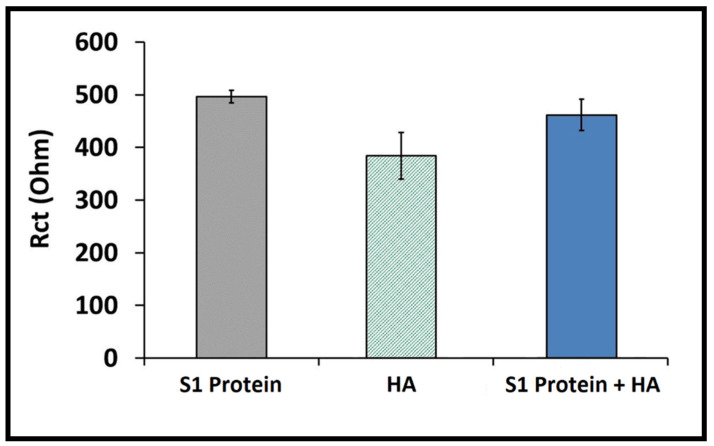
Histograms presenting the data obtained in the selectivity study performed with 1 ng/mL S1 protein, 1 ng/mL HA and the mixture sample containing 1 ng/mL S1 protein and 1 ng/mL HA prepared in diluted artificial saliva (1:20) (*n* = 3).

The lower Rct values were measured in the presence of 1 ng/mL HA protein (384.33 ± 44.09 Ohm; RSD 11.47%, *n* = 3) and compared with those measured in the presence of 1 ng/mL S1 protein and in the mixture sample of S1 protein and HA (1:1) (496.33 ± 12.22 Ohm with an RSD of 2.46%, *n* = 3 and 461.33 ± 30.17 Ohm with an RSD of 6.54%, *n* = 3, respectively) (shown in Figure 6). Therefore, it can be concluded that our impedimetric immunosensor presents a good selectivity toward its target S1 protein over HA, even in a complex sample matrix, e.g., artificial saliva.

In Table 2, an overview of earlier studies presenting impedimetric immunosensors developed for the detection of COVID-19, including this present study, is summarized. The impedimetric immunosensor based on the label-free immunoassay developed herein provides numerous advantages, allowing analysis with a small sample treatment (8 µL) while showing results within a short time (6 min). In addition, there is no need surface modification applied by using metallic nanoparticles or conductive materials in this study in contrast to some of the studies given in Table 2.

There have been many reports in the literature presenting the detection of SARS-CoV-2 virus by different procedures in combination with specific bioreceptors, such as spike protein (S) and nucleocapsid protein (N) [5,47,48]. In addition to the studies targeting spike protein [4,6,7,10,27,49,50], some studies focusing on the detection of S1 subunits [12,51,52] are also presented. The detection of the SARS-CoV-2 virus using combinations of electrochemical techniques, such as voltametric [4,47,48,49,51,53,54], amperometric [5,9,52] and impedimetric [6,7,10,27,55] techniques, was explored for the determination of different bioreceptors of SARS-CoV-2. In comparison to previous studies reporting on the development of different techniques [8,56,57,58,59], the advantages of our label-free impedimetric immunosensor are listed as the simple preparation procedure with short preparation time (i.e., 3 h) and rapid analysis (only 6 min). Moreover, no surface modification using nanomaterials, such as graphene [8,56], graphene oxide [57] or gold nanoparticles [57,58,59], was applied in our study. In contrast to optical detection of SARS-CoV-2 [59,60], our assay is easy to apply without requiring any extra labels (such as fluorescent dye, peptide etc.). Moreover, a lower detection limit was achieved herein, in contrast to the studies of Zhu and Zhou [59], Moncayo et al. [61] and Liu et al. [62].

In contrast to earlier work [5], there is no need to use a secondary antibody in applying the label-free immunoassay, since our assay was mainly based on direct interaction of the antibody with its target without applying any extra intermediaries. Moreover, no surface modification using other molecules such as protein A [6] or biotin [37] on the electrode surface was applied in our study. Overall, the surface treatment/functionalization of the immunosensor was achieved herein straightforwardly and faster than for previous approaches using the indium tin oxide (ITO) electrode [10,28] and the boron-doped polycrystalline diamond electrode [37].

## 4. Conclusions

The impedimetric immunosensor based on the label-free immunoassay for rapid and sensitive detection of the spike S1 protein of SARS-CoV-2 was introduced for the first time in this study. Under optimized conditions, the detection limit was found to be 0.23 ng/mL (equal to 23.92 amol in 8 µL sample) in the linear concentration range of 0.5–10 ng/mL S1 protein in buffer medium. More sensitive results with a lower detection limit (i.e., 0.09 ng/mL, equal to 9.36 amol in 8 µL sample) were achieved in artificial saliva medium with linear concentration range of spike S1 protein from 0.1 to 1 ng/mL. In the selectivity study performed in the artificial saliva medium, the impedimetric immunosensor presented a highly selective response toward its target spike S1 protein of SARS-CoV-2 over influenza hemagglutinin antigen (HA).

In the present work, lower DL values were achieved for electrochemical detection of SARS-CoV-2 in contrast to earlier studies performed by EIS technique [29,30,32,35], voltammetric methods in combination with labeling of alkaline phosphatase enzyme [4], or using a ferri/ferrocyanide indicator [51]. By comparison earlier studies [6,7,10,27,28,29,31,33,36,37], the procedure followed from the preparation of the immunosensor up to measurement of the response in the detection of S1 protein was completed in 3 h, while the impedimetric measurement was performed within only 6 min.

The developed impedimetric immunosensor is disposable, easy to use and cost effective, and does not require trained personnel, thus having significant advantages over traditional analysis methods while allowing individuals to perform tests on their own.

The importance of widespread testing in the COVID-19 pandemic has been noted by many authorities. In addition, there are portable biosensors reported in the literature [7,33] that have been implemented to real samples for the impedimetric detection of COVID-19. In this context, it can be concluded that our impedimetric immunosensor presents a great potential for the development of point-of-care testing.

In conclusion, an impedimetric immunosensor specific to the spike S1 protein of SARS-CoV-2 was developed successfully in our study, and provides a sensitive, selective, fast and precise analysis of S1 protein based on label-free immunoassay. Additionally, our immunoassay procedure in combination with the impedimetric technique could be easily adaptable for the further development of a portable point-of-care test system that may be used in possible pandemics in the future.

## Figures and Tables

**Table 1:** Optimum conditions of the label-free impedimetric immunosensor developed for the detection of spike S1 protein of SARS-CoV-2.

Parameters	Experimental Conditions	Selected Value
**Cab-S1 concentration (µg/mL)**	0.5-1-2-6	1
**BSA concentration (µg/mL)**	0.25-0.5-2-50	0.5
**BSA blocking time (min)**	30-60	30
**Antigen incubation time (min)**	15-30-60	30
**Antigen incubation temperature**	Room temperature-37 °C	37 °C

**Table 2:** Impedimetric immunosensors developed for the detection of SARS-CoV-2.

Analyte	Biorecognition Element	Electrode	Single-Use	Preparation Time	Assay Time	LOD	Application	Reference
SARS-CoV-2 spike protein	IgG anti-SARS-CoV-2 spike antibody	Modified screen-printed electrode with Cu_2_O nanocube	✓	~11 h 40 min	~20 min	0.04 fg/mL	Saliva, artificial nasal and clinical samples	[6]
SARS-CoV-2 spike protein	Angiotensin-converting enzyme-2	Screen-printed paper electrode	✓	~4 h 30 min	~4 min	2.18 fg/mL in PBS solution 1.39 pg/mL in neat saliva	Swab (nose, throat)/saliva samples	[7]
SARS-CoV-2 antibody	Spike RBD protein	Interdigitated electrodes fused to polyethylene terephthalate	x	~1–3 days	−	−	Clinical samples	[27]
SARS-CoV-2 spike RBD protein	Anti-RBD antibodies	GNPs@MUA decorated ITO platform	✓	~13 h 40 min	−	0.58 fg/mL	Artificial nasal secretion samples	[10]
SARS-CoV-2 spike RBD protein	Anti-SARS-CoV-2 spike glycoprotein S1 antibody	Reduced graphene oxide/Glassy carbon electrode	x	~3 h 30 min	−	150 ng/mL	Saliva samples	[29]
SARS-CoV-2 spike RBD protein	SARS-CoV-2 spike S1 Antibody	Electrochemical biochip	✓	~2 h 50 min	<30 min	15 ng/mL	SARS-CoV-2 pseudovirus	[30]
SARS-CoV-2 spike RBD protein	Anti-RBD antibodies	Conducting nanocomposite modified ITO	✓	~3 h 45 min	−	0.58 fg/mL	Nasal secretions	[28]
SARS-CoV spike protein	Anti-SARS-CoV S protein antibodies	Gold-modified screen-printed carbon electrode	✓	~6 h 15 min	~35 min	3.16 pmol/L (83.7 pg/mL)	Saliva sample	[31]
SARS-CoV-2 spike protein	Peptide	Screen-printed gold electrode	✓	~1 h 55 min	~15 min	18.2 ng/mL	Clinical samples (nasopharyngeal swabs samples)	[32]
SARS-CoV-2 virus	Virus-imprinted sensor (VIP)	Carbon nanotube/ Tungsten Oxide/Screen-printed carbon electrode	✓	~3 h 50 min	−	57 pg/mL	Clinical samples (oropharyngeal and/or nasopharyngeal swab)	[33]
SARS-CoV-2 antibody	SARS-CoV-2 nucleoprotein	Gold nanoparticles modified poly (3,4-ethylenedioxythiophene)	✓	~1 h	~30 min	−	Serum samples	[34]
SARS-CoV-2 spike protein	Angiotensin-converting enzyme-2 (ACE2) and cluster of differentiation 147 (CD147)	Gold screen-printed electrode	✓	~3 h	~5 min	299.30 ng/mL for ACE2 38.99 ng/mL for CD147	Oropharyngeal and/or nasopharyngeal swab	[35]
SARS-CoV-2 spike protein	SARS-CoV-2 spike protein antibody	Interdigitated gold electrode	✓	~Over 48 h	−	0.179 fg/mL	Inactivated SARS-CoV-2 virus	[36]
SARS-CoV-2 spike S1 protein (S1 protein)	Anti-SARS-CoV-2 S1 antibody	Screen-printed carbon electrode	✓	~3 h	~6 min	0.23 ng/mL (equals to 23.92 amol in 8 µL sample) in buffer medium 0.09 ng/mL (equals to 9.36 amol in 8 µL sample) in artificial saliva	Artificial saliva samples	This study

## Data Availability

The data presented in this study are available within the article. Other data that support the findings of this study are available upon request from the corresponding author as well as co-authors.

## Supplementary Materials

The following supporting information can be downloaded at: https://www.mdpi.com/article/10.3390/diagnostics12081992/s1, Table S1. The information about antibodies and proteins used in this study. Table S2. The average values of anodic current (**I_a_**) and cathodic current (**I_c_**) with anodic/cathodic charge transfer values (**Q_a/_Q_c_**) with the values of ΔEp measured in redox probe by using CV in combination with each of electrodes; SPE, SPE/EDC-NHS, SPE/EDC-NHS/Cab-S1, SPE/EDC-NHS/Cab-S1/BSA and SPE/EDC-NHS/Cab-S1/BSA/S1 Protein. Table S3. The average Rct value with the difference ratio (%) obtained before and after blocking step in experimental procedures by using human monoclonal, rabbit monoclonal and rabbit polyclonal capture antibodies as Cab-S1. Table S4. Average Rct values with the difference ratio (%) at immunosensor response in the presence of Cab-S1, BSA and S1 protein at different Cab-S1 concentrations. Table S5. Average Rct values with the difference ratio (%) at immunosensor response in the presence of Cab-S1, BSA and S1 protein at different BSA concentrations. Table S6. Average Rct values with the difference ratio (%) at immunosensor response in the presence of Cab-S1, BSA and S1 protein at different BSA blocking times. Table S7. Average Rct values with the difference ratio (%) at immunosensor response in the presence of Cab-S1, BSA and S1 protein at different S1 protein incubation times. Table S8. Average Rct values with the difference ratio (%) at immunosensor response in the presence of Cab-S1, BSA and S1 protein at different S1 protein incubation temperatures. Table S9. The average Rct values (*n* = 6) with RSD (%) obtained in presence of S1 protein in different concentrations. Figure S1. Images of the immunosensor with test setup. (1) Top view of the test setup, (2) side view of the test setup. Figure S2. Cyclic voltammograms of SPE, SPE/EDC-NHS, SPE/EDC-NHS/Cab-S1, SPE/EDC-NHS/Cab-S1/BSA and SPE/EDC-NHS/Cab-S1/BSA/S1 Protein measured in 5 mM [Fe(CN)_6_]^3−/4−^ containing 0.1 M KCl by following experimental conditions: 5/8 mM EDC/NHS, 1 µg/mL Cab-S1, 10 µg/mL BSA and 1 µg/mL S1 protein and the potential range between −0.5 V and +1.0 V with a scan rate: 50 mV/s. Figure S3. The effect of each experimental parameters upon to the response of impedimetric immunosensor: (A) Cab-S1 concentration, (B) BSA concentration, (C) BSA blocking time, (D) S1 protein incubation time, (E) S1 protein incubation temperature. White columns represent after the immobilization of Cab-S1, blue columns represent after the blocking with BSA, green columns represent after the incubation of S1 protein. Figure S4. Line graph representing the average Rct values (*n* = 3) in the concentration range of S1 protein in buffer medium. Figure S5. Nyquist diagrams obtained in the presence of S1 protein prepared in the artificial saliva sample diluted in various ratios. (a) 1:20 diluted, (b) 1:5 diluted artificial saliva medium (control experiment), 0.5 ng/mL S1 protein prepared in (c) 1:5 diluted, (d) 1:20 diluted artificial saliva medium. Figure S6. Line graph representing the average Rct values (*n* = 3) in the concentration range of S1 protein in artificial saliva medium. Figure S7. Nyquist diagrams obtained in the selectivity study performed with (a) 1 ng/mL S1 protein, (b) 1 ng/mL HA and (c) the mixture of 1 ng/mL S1 protein and 1 ng/mL HA prepared in 1:20 artificial saliva medium (*n* = 3). References [63,64,65,66,67] are cited in Supplementary Materials.
