# Supplementary material for: Impedimetric Detection Based on Label-Free Immunoassay Developed for Targeting Spike S1 Protein of SARS-CoV-2
Source: Diagnostics (Basel). 2022 Aug 17;12(8):1992. doi: 10.3390/diagnostics12081992 (PMC9407092; doi:10.3390/diagnostics12081992)
Supplement: Supplementary file 1 [file diagnostics-12-01992-s001.zip › diagnostics-1809077-supplementary.pdf]

diagnostics

Article

Impedimetric Detection Based on Label-Free Immunoassay Developed for Targeting Spike S1 Protein of SARS-CoV-2

Arzum Erdem *, Huseyin Senturk, Esma Yildiz and Meltem Maral

Analytical Chemistry Department, Faculty of Pharmacy, Ege University, Bornova 35100, Izmir, Turkey
* Correspondence: arzum.erdem@ege.edu.tr or arzume@hotmail.com

Chemicals and apparatus

The information about SARS-CoV-2 spike S1 protein, capture antibodies (Cab-S1), Hemagglutinin antigen (HA) and Bovine serum albumin (BSA) was given in Table S1.

Table S1. The information about antibodies and proteins used in this study.

Antibody	COMPANY
rabbit monoclonal anti-SARS-CoV-2 S1 antibody	ProSci, (Cat. Number: 10-350)
rabbit polyclonal anti-SARS-CoV-2 S1 antibody	ProSci, (Cat. Number: 9083)
human monoclonal anti-SARS-CoV-2 S1 antibody	MyBioSource, (Cat. Number: MBS8574745)
**Protein**	**COMPANY**
SARS-CoV-2 S1 protein	ProSci, (Cat. Number: 97-087)
Bovine serum albumin (BSA)	Sigma-Aldrich, (Cat. Number: A9056)
Influenza Hemagglutinin antigen (HA)	Sigma-Aldrich, (Cat. Number: I2149)

Phosphate buffer solution (PBS, 50 mM, pH 7.4) was used to prepare diluted solutions of antibodies and antigens. Acetate buffer solution (ABS, 0.5 M, pH 4.8) was used for the electrochemical activation of electrode. Artificial Saliva was purchased from Pickering Laboratories. Other chemicals were used in analytical reagent grade and were supplied from Sigma-Aldrich and Merck.

All electrochemical measurements were performed via AUTOLAB 204 PGSTAT (FRA 32M) instrument NOVA 1.11 software (Eco Chemie, The Netherland) in a faraday cage. The Ref. DSC connector (Metrohm DropSens, Spain) was used to provide the connection between the electrode and the device. The planar screen-printed electrode (Metrohm Dropsens Ref. 110); SPE in 3.3 × 1.0 × 0.05 cm (length × width × height) consists of three main parts, which are carbon working electrode (4 mm in diameter), a carbon counter electrode and a silver reference electrode.

The images of the immunosensor with test setup was shown in Figure S1.

Citation: Erdem, A.; Senturk, H.; Yildiz, E.; Maral, M. Impedimetric Detection Based on Label-Free Immunoassay Developed for Targeting Spike S1 Protein of SARS-CoV-2. *Diagnostics* **2022**, *12*, 1992. https://doi.org/10.3390/diagnostics12081992

Academic Editor: Xavier Muñoz-Berbel

Received: 24 June 2022
Accepted: 12 August 2022
Published: 17 August 2022

Publisher's Note: MDPI stays neutral with regard to jurisdictional claims in published maps and institutional affiliations.

Copyright: © 2022 by the authors. Submitted for possible open access publication under the terms and conditions of the Creative Commons Attribution (CC BY) license (https://creativecommons.org/licenses/by/4.0/).

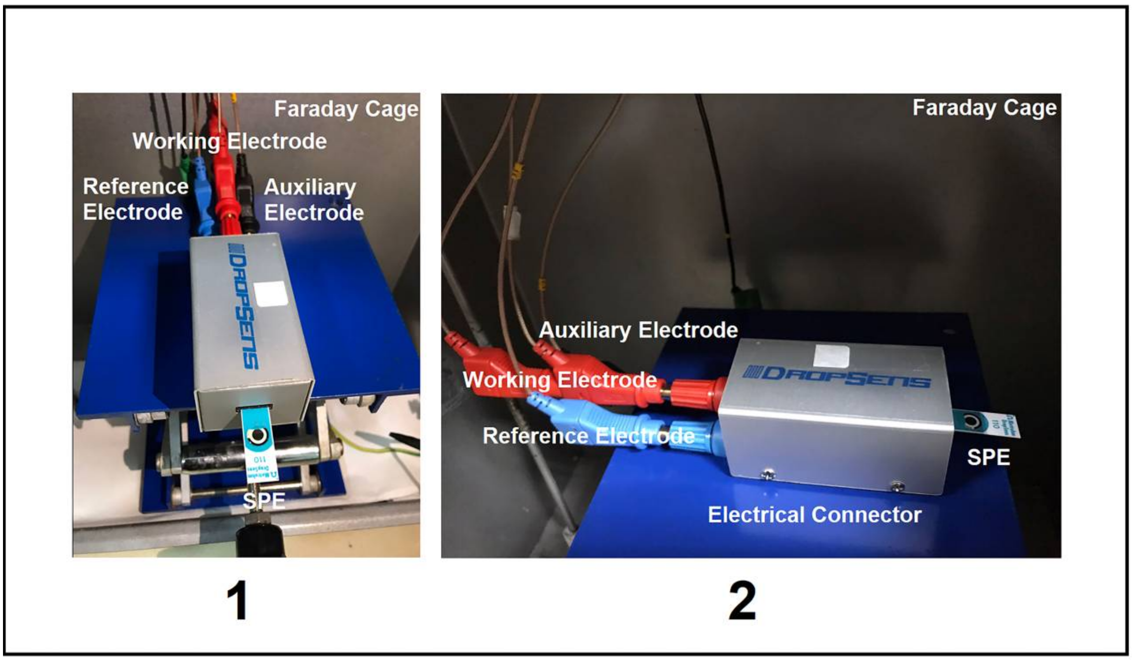

Figure S1. Images of the immunosensor with test setup. (1) Top view of the test setup, (2) side view of the test setup.

The equivalent circuit model (Randles circuit) is used to fit the data obtained by impedimetric measurments, which consists of charge transfer resistance (Rct), solution resistance (Rs), Warburg impedance (W), constant phase element (C). The respective semicircle diameter corresponds to Rct. The related circuit model is used in order to fit the impedance data as shown in all figures containing Nyquist diagrams that are plotted by using the data obtained by EIS measurements. All data presented in each Nyquist diagram is the fitted version of the related data.

Characterization on the construction of immunosensor for impedimetric detection of S1 Protein

The characterization on the construction of our immunosensor was carried out using cyclic voltammetry (CV) technique in the solution of redox probe, and accordingly, the results were given in Figure S2 and Table S2.

CV experiments were performed by using 5/8 mM EDC/NHS, 1 µg/mL Cab-S1, 10 µg/mL BSA and 1 µg/mL S1 protein and accordingly, the procedure was followed as mentioned above. CV measurement was performed in the potential range from −0.5 V to +1.0 V with the scan rate as 50 mV/s in the solution of redox probe containing 5 mM $[Fe(CN)_6]^{3-/4-}$ prepared in PBS (50 mM, pH 7.40) with 0.1 M KCl. 40 µL of redox probe solution was pipetted onto the electrode surface and then, CV measurement was performed.

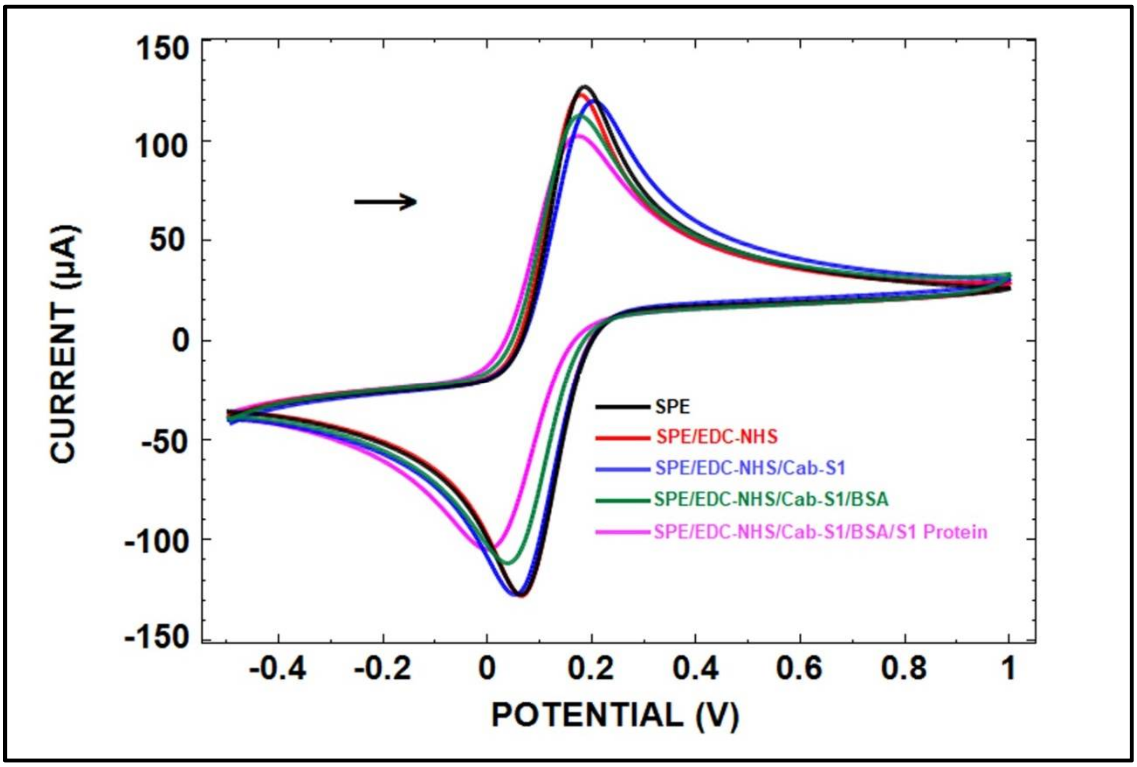

Figure S2. Cyclic voltammograms of SPE, SPE/EDC-NHS, SPE/EDC-NHS/Cab-S1, SPE/EDC-NHS/Cab-S1/BSA and SPE/EDC-NHS/Cab-S1/BSA/S1 Protein measured in 5 mM $[Fe(CN)_6]^{3-/4-}$ containing 0.1 M KCl by following experimental conditions: 5/8 mM EDC/NHS, 1 µg/mL Cab-S1, 10 µg/mL BSA and 1 µg/mL S1 protein and the potential range between −0.5 V and +1.0 V with a scan rate: 50 mV/s.

Table S2. The average values of anodic current **(I_a)** and cathodic current **(I_c)** with anodic/cathodic charge transfer values (**Q_a/ Q_c**) with the values of ΔEp measured in redox probe by using CV in combination with each of electrodes; SPE, SPE/EDC-NHS, SPE/EDC-NHS/Cab-S1, SPE/EDC-NHS/Cab-S1/BSA and SPE/EDC-NHS/Cab-S1/BSA/S1 Protein.

	**I_a (nA)**	**I_c (nA)**	**ΔEp (mV)**	**Q_a (mC)**	**Q_c (mC)**
**SPE**	136,270.00 ± 2913.28	123,630.00 ± 2715.29	120	1.26 ± 0.01	1.13 ± 0.01
**SPE/EDC-NHS**	126,015.00 ± 4589.12	119,105.00 ± 4744.69	123	1.23 ± 0.01	1.08 ± 0.03
**SPE/EDC-NHS/Cab-S1**	124,355.00 ± 5946.77	120,240.00 ± 6957.93	113	1.28 ± 0.05	1.15 ± 0.04
**SPE/EDC-NHS/Cab-S1/BSA**	123,135.00 ± 3528.46	111,000.00 ± 3917.37	132	1.24 ± 0.01	1.12 ± 0.01
**SPE/EDC-NHS/Cab-S1/BSA/S1 Protein**	112,900.00 ± 3790.09	101,865.50 ± 3867.17	142	1.23 ± 0.01	1.06 ± 0.02

Optimization of assay parameters

Overall of optimization studies, the difference ratio (%) was calculated according to Equation (S1) given below. In the optimization studies, the calculation was performed according to results obtained by two repetitive measurements.

$$\textbf{Difference ratio}\ (\%) = \frac{Rct_1 - Rct_2}{Rct_2} \times 100 \qquad \textbf{(S1)}$$

Rct_1 is average Rct value after the incubation of S1 protein, Rct_2 is average Rct value before the incubation of S1 protein. First, SARS-CoV-2 S1 human monoclonal antibody (mAb) was immobilized as capture antibody (Cab-S1) onto the chemical activated surface. The chemical activation was performed by following the procedure of EDC/NHS chemistry [1,2]. After antibody immobilization onto electrode surface, it was blocked by using BSA in order to prevent non-specific binding of any molecules at electrode surface. Then, spike S1 protein was added on the SPE surface and allowed to interact with antibody. Accordingly, immunocomplex formation was achieved due to the high affinity between antigen and antibody. In our study, antigen specificity was increased by means of the mAb as reported in earlier work [3].

The selection of antibodies sources is a critical issue in aspects of development of immunoassay in a good selectivity with a high sensitivity for analysis of antigens. In our study, anti-SARS-CoV-2 antibodies such as, human monoclonal, rabbit monoclonal and rabbit polyclonal were used as capture antibody (Cab-S1). Accordingly, the immunoreaction response was investigated in terms of its capturing target antigen; SARS-CoV-2 S1 protein. To examine the selection of antibodies sources, a batch of experiments was performed by using 1 µg/mL Cab-S1 with 1 hour immobilization time, 50 µg/mL BSA with 1 hour blocking time and 0.5 µg/mL SARS-CoV-2 S1 protein with 1 hour incubation time. The highest difference ratio (15.39 % increase) was recorded at the average Rct with human monoclonal antibody (Table S3). Consequently, the procedure was followed in our study by using human monoclonal antibody as Cab-S1.

Table S3. The average Rct value with the difference ratio (%) obtained before and after blocking step in experimental procedures by using human monoclonal, rabbit monoclonal and rabbit polyclonal capture antibodies as Cab-S1.

Capture Antibody (Cab-S1)	$R_{ct(Cab\text{-}S1)}$ (Ohm)	$R_{ct(BSA)}$ (Ohm)	$R_{ct(S1\ protein)}$ (Ohm)	Difference ratio (%)
**Human monoclonal antibody**	**178.93 ± 23.86**	**579.33 ± 52.13**	**668.50 ± 93.18**	**15.39 increase**
**Rabbit monoclonal antibody**	206.25 ± 47.02	556.50 ± 119.50	600.00 ± 147.08	7.82 increase
**Rabbit polyclonal antibody**	390.00 ± 77.78	928.00 ± 73.54	1015.00 ± 7.07	9.38 increase

In our study, the EDC/NHS concentration was chosen as 5mM EDC/ 8 mM, according to previous reported studies [1,2].

The optimization of Cab-S1 concentration was investigated in range of 0.5–6 μg/mL. The experimental procedure was performed using Cab-S1 in different concentrations (immobilization time, 1 hour), 2 μg/mL BSA (blocking time, 1 hour) and 0.5 μg/mL SARS-CoV-2 S1 protein (incubation time, 1 hour). The optimum Cab-S1 concentration was chosen as 1 μg/mL due to highest difference ratio (14.49 %) in the presence of S1 protein resulting with more repeatable results (shown in Figure S3A and Table S4).

In the presence of 1 μg/mL Cab-S1, BSA concentration was examined in various concentrations; 0.25-0.5-2-50 μg/mL. The experimental procedure was followed using 1 μg/mL Cab-S1 (immobilization time, 1 hour), BSA in different concentrations (blocking time, 1 hour) and 0.5 μg/mL SARS-CoV-2 S1 protein (incubation time, 1 hour). After the blocking step performed in the presence of 0.5 μg/mL BSA, the highest difference ratio (14.78 %) was obtained. Therefore, 0.5 μg/mL was chosen as optimum BSA concentration (Figure S3B and Table S5).

BSA blocking time was then optimized in this following conditions: 1 μg/mL Cab-S1 (immobilization time, 1 hour), 0.5 μg/mL BSA (blocking time, 30-60 minutes) and 0.5 μg/mL SARS-CoV-2 S1 protein (incubation time, 1 hour). After 30 minutes blocking step, the highest difference ratio (16.73 %) was obtained. Hence, the optimum BSA blocking time was chosen as 30 minutes (Figure S3C and Table S6).

The effect of antigen incubation time upon to immunosensor response was investigated by following conditions: 1 μg/mL Cab-S1 (immobilization time, 1 hour), 0.5 μg/mL BSA (blocking time, 30 minutes) and 0.5 μg/mL SARS-CoV-2 S1 protein (incubation time, 15-30-60 minutes). The highest difference ratio (20.95 %) was obtained in 30 minutes antigen incubation time (Figure S3D and Table S7). Therefore, the optimum antigen incubation time was chosen as 30 minutes.

Electrochemical response of immunosensor depending on temperature was examined at room temperature and 37°C. In contrast to results obtained at room temperature (20.95 %), a remarkable increase (25.31 %) at the average Rct value was achieved at 37°C. Similarly to the results reported in previous studies [4–6], the optimum temperature for S1 protein incubation was chosen as 37°C (Figure S3E and Table S8).

In brief, the experimental procedure was constituted herein under the optimum conditions (shown in Table 1) as follows: EDC/NHS: 5mM/8mM, Cab-S1: 1 μg/mL, BSA: 0.5 μg/mL, BSA incubation time: 30 min, antigen incubation time: 30 min, antigen incubation temperature: 37°C.

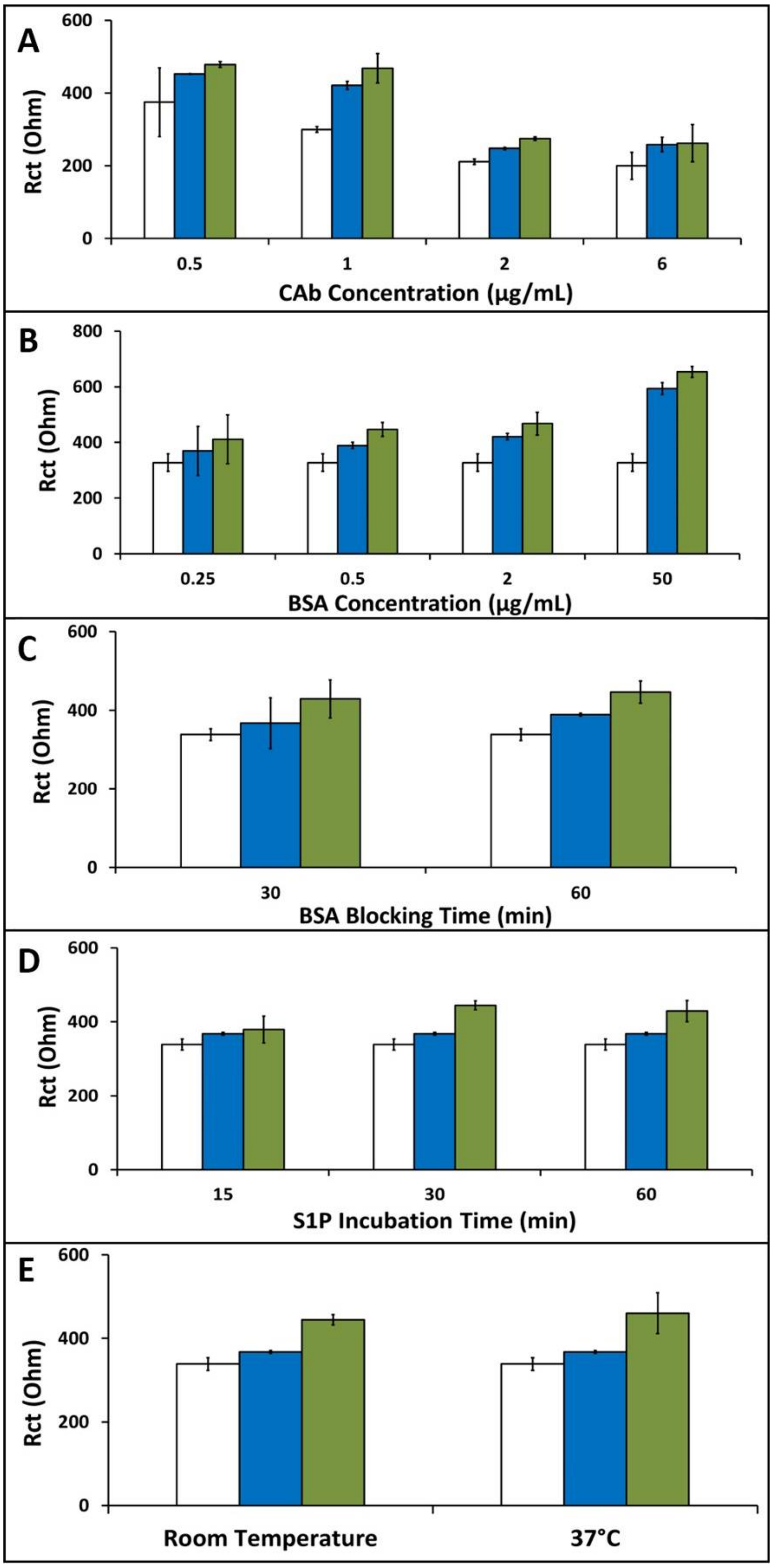

Figure S3. The effect of each experimental parameters upon to the response of impedimetric immunosensor: (A) Cab-S1 concentration, (B) BSA concentration, (C) BSA blocking time, (D) S1 protein incubation time, (E) S1 protein incubation temperature. White columns represent after the immobilization of Cab-S1, blue columns represent after the blocking with BSA, green columns represent after the incubation of S1 protein.

Table S4. Average Rct values with the difference ratio (%) at immunosensor response in the presence of Cab-S1, BSA and S1 protein at different Cab-S1 concentrations.

**Cab-S1 Concentration (μg/mL)**	**$R_{ct(Cab-S1)}$ (Ohm)**	**$R_{ct(BSA)}$ (Ohm)**	**$R_{ct(S1\ protein)}$ (Ohm)**	**Difference ratio (%)**
**0.5**	374.50 ± 94.05	452.50 ± 0.71	478.50 ± 7.78	5.75 increase
**1**	**299.50 ± 7.78**	**421.00 ± 11.31**	**467.67 ± 40.62**	**11.08 increase**
**2**	211.00 ± 7.07	247.50 ± 3.54	274.50 ± 4.95	10.91 increase
**6**	199.50 ± 37.48	258.00 ± 19.80	262.00 ± 50.91	1.55 increase

Table S5. Average Rct values with the difference ratio (%) at immunosensor response in the presence of Cab-S1, BSA and S1 protein at different BSA concentrations.

**BSA Concentration (μg/mL)**	**$R_{ct(Cab-S1)}$ (Ohm)**	**$R_{ct(BSA)}$ (Ohm)**	**$R_{ct(S1\ protein)}$ (Ohm)**	**Difference ratio (%)**
**0.25**	**327.50 ± 31.82**	369.50 ± 88.39	411.00 ± 87.68	11.23 increase
**0.5**		**389.00 ± 11.31**	**446.50 ± 24.75**	**14.78 increase**
**2**		421.00 ± 11.31	467.67 ± 40.62	11.08 increase
**50**		594.00 ± 21.21	653.50 ± 19.09	10.02 increase

Table S6. Average Rct values with the difference ratio (%) at immunosensor response in the presence of Cab-S1, BSA and S1 protein at different BSA blocking times.

**BSA Blocking Time (min)**	**$R_{ct(Cab-S1)}$ (Ohm)**	**$R_{ct(BSA)}$ (Ohm)**	**$R_{ct(S1\ protein)}$ (Ohm)**	**Difference ratio (%)**
**30**	**338.50 ± 14.85**	**367.50 ± 3.54**	**429.00 ± 28.28**	**16.73 increase**
**60**		389.00 ± 11.31	446.50 ± 24.75	14.78 increase

Table S7. Average Rct values with the difference ratio (%) at immunosensor response in the presence of Cab-S1, BSA and S1 protein at different S1 protein incubation times.

S1 protein incubation time(min)	$R_{ct(Cab\text{-}S1)}$ (Ohm)	$R_{ct(BSA)}$ (Ohm)	$R_{ct(S1\ protein)}$ (Ohm)	Difference ratio (%)
15	338.50 ± 14.85	367.50 ± 3.54	379.50 ± 36.06	3.27 increase
30			**444.50 ± 12.02**	**20.95 increase**
60			429.00 ± 28.28	16.73 increase

Table S8. Average Rct values with the difference ratio (%) at immunosensor response in the presence of Cab-S1, BSA and S1 protein at different S1 protein incubation temperatures.

S1 protein incubation temperature	$R_{ct(Cab\text{-}S1)}$ (Ohm)	$R_{ct(BSA)}$ (Ohm)	$R_{ct(S1\ protein)}$ (Ohm)	Difference ratio (%)
37 °C	338.50 ± 14.85	367.50 ± 3.54	**460.50 ± 48.79**	**25.31 increase**
Room Temperature			444.50 ± 12.02	20.95 increase

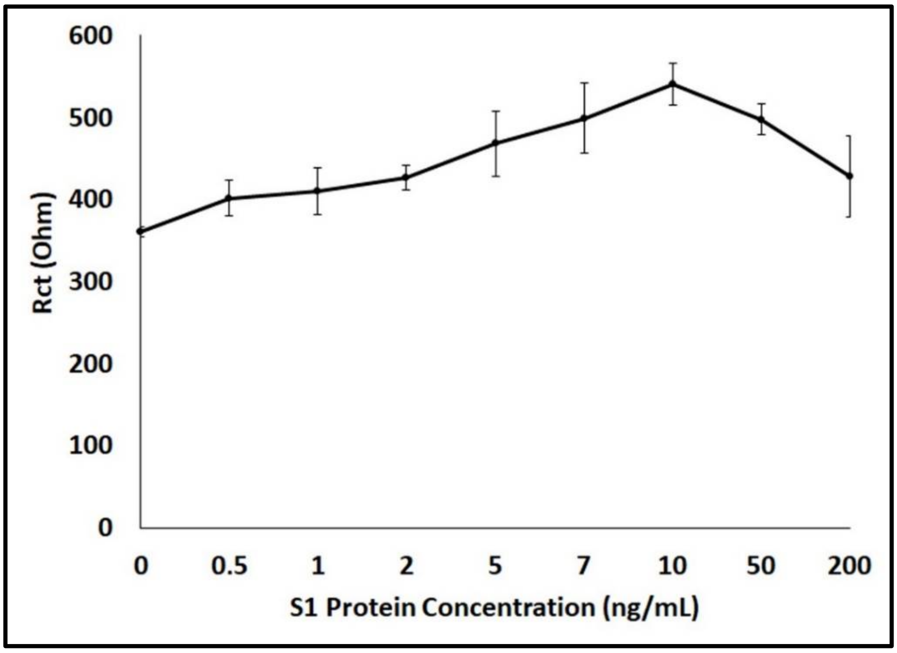

Figure S4. Line graph representing the average Rct values (n=3) in the concentration range of S1 protein in buffer medium.

The line graph based on Rct values obtained in the range of S1 protein from 0 ng/mL up to 200 ng/mL is presented in Fig S4. When the lowest S1 protein concentration value (0.5 ng/mL) of this range was incubated on the electrode surface, a significant increase was recorded in comparison to the Rct value measured in the absence of S1 Protein.

At higher concentrations of of S1 Protein than 10 ng/mL, a decrease at Rct value was recorded. For instance, the lowest Rct value was obtained in the absence of S1 Protein of all when the concentration of S1 Protein is increased as 5 folds or 20 folds (from 10 to 50 ng/mL or 200 ng/mL).

Table S9. The average Rct values (n=6) with RSD (%) obtained in presence of S1 protein in different concentrations.

S1 protein Concentration (ng/mL)	Rct values (Ohm)	RSD %
**0.5**	401.67 ± 22.12	5.51
**1**	410.50 ± 28.32	6.90
**2**	426.33 ± 15.04	3.53
**5**	468.17 ± 39.57	8.45
**7**	499.33 ± 42.21	8.45
**10**	541.17 ± 25.51	4.71

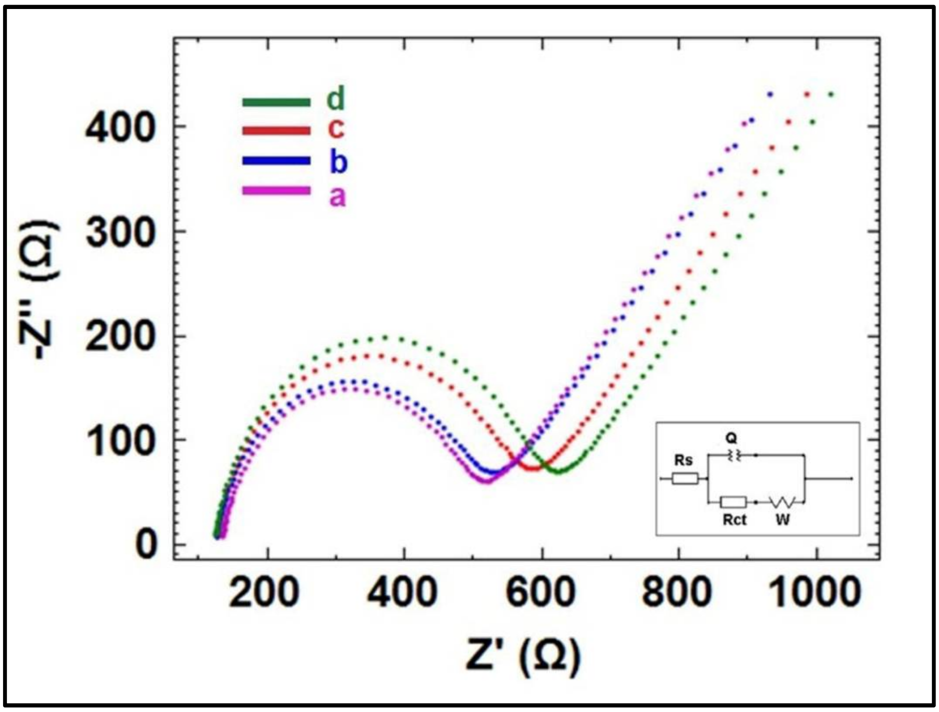

Figure S5. Nyquist diagrams obtained in the presence of S1 protein prepared in the artificial saliva sample diluted in various ratios. (a) 1:20 diluted, (b) 1:5 diluted artificial saliva medium (control experiment), 0.5 ng/mL S1 protein prepared in (c) 1:5 diluted, (d) 1:20 diluted artificial saliva medium.

The difference ratio (%) was calculated according to Equation (S1) given in Supporting information. Rct_1 is average Rct value after the incubation of 0.5 ng/mL S1 protein in artificial saliva, Rct_2 is average Rct value after incubation of artificial saliva sample (i.e control) in the absence of S1 protein.

The experiments were conducted in our study in the medium of artificial saliva over a wide concentration range of S1 protein. A decrease at Rct value occurred in higher concentration of S1 Protein than 1 ng/mL. Therefore, the experiments were performed in the range of lower concentration values of S1 protein prepared in the diluted medium of artificial saliva. Consequently, the linearity was recorded in the concentration range of 0.1 to 1 ng/mL S1 Protein. The results are shown in Figure S6.

Similar to our study, in the study of Chen et al. [7] as well as our previous study [8], a lower concentration range of target analyte was studied in artificial saliva medium in comparison to the buffer medium by resulting with a lower detection limit.

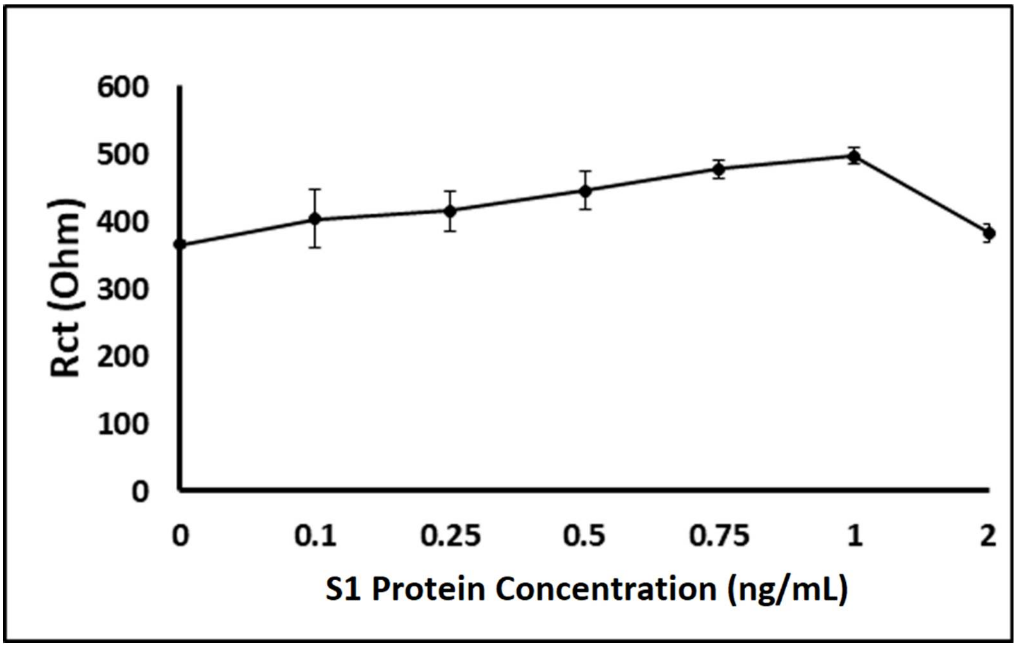

Figure S6. Line graph representing the average Rct values (n=3) in the concentration range of S1 protein in artificial saliva medium.

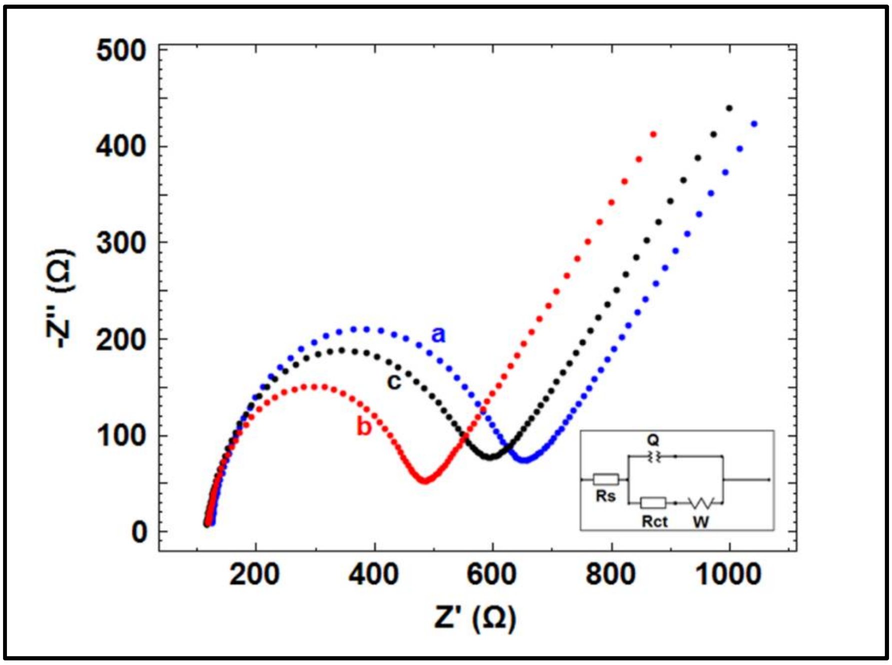

Figure S7. Nyquist diagrams obtained in the selectivity study performed with (a) 1 ng/mL S1 protein, (b) 1 ng/mL HA and (c) the mixture of 1 ng/mL S1 protein and 1 ng/mL HA prepared in 1:20 artificial saliva medium (n=3).

References

1. Lamarca, R.S.; Faria, R.A.D. De; Zanoni, M.V.B.; Nalin, M.; Lima Gomes, P.C.F. De; Messaddeq, Y. Simple, fast and environmentally friendly method to determine ciprofloxacin in wastewater samples based on an impedimetric immunosensor. *RSC Adv.* **2020**, *10*, 1838–1847, doi:10.1039/c9ra09083e.
2. Rocha, C.G.; Ferreira, A.A.P.; Yamanaka, H. Label-free impedimetric immunosensor for detection of the textile azo dye Disperse Red 1 in treated water. *Sensors Actuators, B Chem.* **2016**, *236*, 52–59, doi:10.1016/j.snb.2016.05.040.
3. Ramos-Vara, J.A. Technical aspects of immunohistochemistry. *Vet. Pathol.* **2005**, *42*, 405–426, doi:10.1354/vp.42-4-405.
4. Zhou, J.; Du, L.; Zou, L.; Zou, Y.; Hu, N.; Wang, P. An ultrasensitive electrochemical immunosensor for carcinoembryonic antigen detection based on staphylococcal protein A - Au nanoparticle modified gold electrode. *Sensors Actuators, B Chem.* **2014**, *197*, 220–227, doi:10.1016/j.snb.2014.02.009.
5. Yu, L.; Zhang, Y.; Hu, C.; Wu, H.; Yang, Y.; Huang, C.; Jia, N. Highly sensitive electrochemical impedance spectroscopy immunosensor for the detection of AFB1 in olive oil. *Food Chem.* **2015**, *176*, 22–26, doi:10.1016/j.foodchem.2014.12.030.
6. Chen, X.; Wang, Y.; Zhou, J.; Yan, W.; Li, X.; Zhu, J.J. Electrochemical impedance immunosensor based on three-dimensionally ordered macroporous gold film. *Anal. Chem.* **2008**, *80*, 2133–2140, doi:10.1021/ac7021376.
7. Chen, P.H.; Huang, C.C.; Wu, C.C.; Chen, P.H.; Tripathi, A.; Wang, Y.L. Saliva-based COVID-19 detection: A rapid antigen test of SARS-CoV-2 nucleocapsid protein using an electrical-double-layer gated field-effect transistor-based biosensing system. *Sensors Actuators B Chem.* **2022**, *357*, 131415, doi:10.1016/j.snb.2022.131415.
8. Erdem, A.; Senturk, H.; Yildiz, E.; Maral, M. Amperometric immunosensor developed for sensitive detection of SARS-CoV-2 spike S1 protein in combined with portable device. *Talanta* **2022**, *244*, 123422, doi:10.1016/j.talanta.2022.123422.
